# Recurrent Multi-territorial Ischemic Stroke As the Initial Presentation of Thrombotic Thrombocytopenic Purpura

**DOI:** 10.7759/cureus.79155

**Published:** 2025-02-17

**Authors:** Noah Reichman, Grace Marie Nicole Biso, Hesham Masoud

**Affiliations:** 1 Neurology, State University of New York Upstate Medical University, Syracuse, USA

**Keywords:** cerebrovascular accident (stroke), ischemic stroke, recurrent stroke in young, stroke in the young, thrombotic thrombocytopenic purpura, thrombotic thrombocytopenic purpura (ttp)-like syndrome

## Abstract

Thrombotic thrombocytopenic purpura (TTP) is a rare hypercoagulable disorder characterized by fever, acute hemolytic anemia, thrombocytopenia, neurologic deficits, and renal failure. Due to the rarity of TTP, the infrequency of a complete TTP pentad, variable and atypical presentations, and overlap with other thrombotic microangiopathy, diagnosis is difficult to achieve. Here, we describe a middle-aged patient with recurrent multi-territory strokes, with the development of thrombocytopenia occurring later in the course of her illness without fever, hemolytic anemia, or renal failure. Etiologies for prior history of ischemic stroke were confounded by the presence of intrinsic cerebral arteriopathy at the bilateral anterior cerebral artery, middle cerebral artery, and posterior cerebral artery territories attributed to accelerated atherosclerosis from concurrent tobacco smoking and marijuana abuse. Extensive workup also disclosed inter-atrial shunt (grade III patent foramen ovale), which was subsequently treated with device closure for presumed secondary stroke prevention. Due to the development of thrombocytopenia and recurrent multi-territory strokes, an ADAMTS13 (a disintegrin and metalloprotease with a thrombospondin type 1 motif, member 13) screen was ordered and was positive. The ADAMTS13 activity was <5%, while the ADAMTS13 inhibitor Bethesda titer was notably high (1.4, normal <0.4). For acute therapy, a three-day 1 mg/kg methylprednisolone was started, and a hematology service was consulted for co-management. The patient completed four rounds of plasmapheresis while receiving 90 mg of prednisone daily. She was then started on a regimen to complete four doses of weekly rituximab. The patient improved clinically during her stay, with noted improvements in platelet count and ADAMTS13 activity. In conclusion, thrombocytopenia may appear until later in the disease course with variant presentations of TTP. A low threshold to consider atypical etiologies when pursuing workup for cryptogenic stroke should be in mind when evaluating young adults with recurrent multi-territory ischemic stroke.

## Introduction

Thrombotic thrombocytopenic purpura (TTP) is a rare and potentially fatal thrombotic microangiopathy characterized by the pentad of fever, hemolytic anemia, thrombocytopenia, renal failure, and fluctuating neurological changes [1]. TTP has an annual prevalence of 10 cases per million people and an annual incidence of one new case per million people. It tends to affect otherwise healthy adults with a mean age of 49 (in the United States), females, and African American individuals compared to White American individuals. Although the disease often presents for the first time during adulthood, one in ten cases occurs in children [2].

The pathogenesis of TTP involves a severe deficiency of ADAMTS13 (a disintegrin and metalloprotease with a thrombospondin type 1 motif, member 13), a zinc-containing metalloprotease mainly produced in the liver. This enzyme cleaves von Willebrand factor (vWF), a multimeric glycoprotein with the dual functions of mediating platelet adhesion and aggregation to sites of damaged endothelium, as well as acting as the carrier protein for factor VIII (FVIII), shielding it from proteolytic breakdown. Defective ADAMTS13 accumulates large vWF molecules, leading to platelet adhesion, aggregation, and thrombosis [3,4]. This enzyme deficiency stems from either mutation of the ADAMTS13 gene (hereditary TTP) or autoantibodies against ADAMTS13 (acquired or idiopathic TTP).

Hereditary TTP, known as Upshaw-Shulman syndrome, accounts for only 2-4% of all cases. This form typically manifests after an acute illness or during pregnancy, whereas acquired TTP has been linked to HIV, antiplatelets and immunosuppressive agents, pregnancy, and estrogen-containing birth control [5].

Due to the rarity of TTP, the infrequency of a complete TTP pentad, variable and atypical presentations, and overlap with other thrombotic microangiopathies (TMAs), diagnosis is difficult to achieve. The current approach is to determine a presumptive diagnosis based on clinical prediction tools (PLASMIC score) and ADAMTS13 activity. The PLASMIC score allocates one point each for platelet count <30 x 109/L, evidence of hemolysis (elevated reticulocyte count, elevated indirect bilirubin, or decreased haptoglobin), absence of active cancer, no history of solid organ or stem cell transplantation, international normalized ratio (INR) <1.5, and serum creatinine <2 mg/dL. An intermediate to high-risk PLASMIC score (5-7) is enough to start life-saving therapeutic plasma exchange (TPE) while waiting for ADAMTS13 activity, which usually takes days to result. Our patient's PLASMIC score is 4 for no active cancer, no history of solid-organ or stem-cell transplant, INR <1.5, and creatinine <2.0 mg/dL. Hence, treatment started after ADAMTS13 results. ADAMTS13 activity <10% confirms TTP. However, ADAMTS13 activity <10% is neither 100% sensitive nor 100% specific. Patients with TTP can have ADAMTS13 activity >10%. Meanwhile, ADAMTS13 activity <10% can be seen in conditions such as sepsis, hepatic necrosis, HIV with Kaposi sarcoma, and graft-versus-host disease [3,6,7].

The abstract was presented at the 2023 Society of Vascular and Interventional Neurology’s (SVIN) Annual Meeting on November 16-18, 2023.

## Case presentation

**Figure 1:**
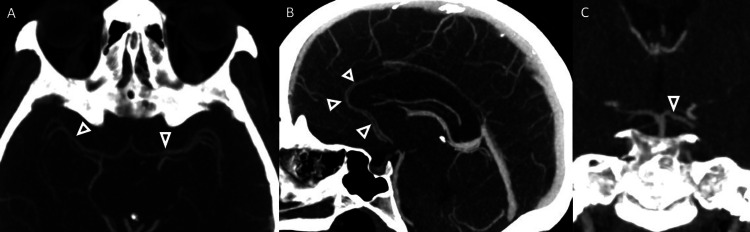
Arteriopathy (arrowheads) on MCA (A), ACA (B), and PCA (C) MCA: middle cerebral artery, ACA: anterior cerebral artery, PCA: posterior cerebral artery

**Figure 2:**
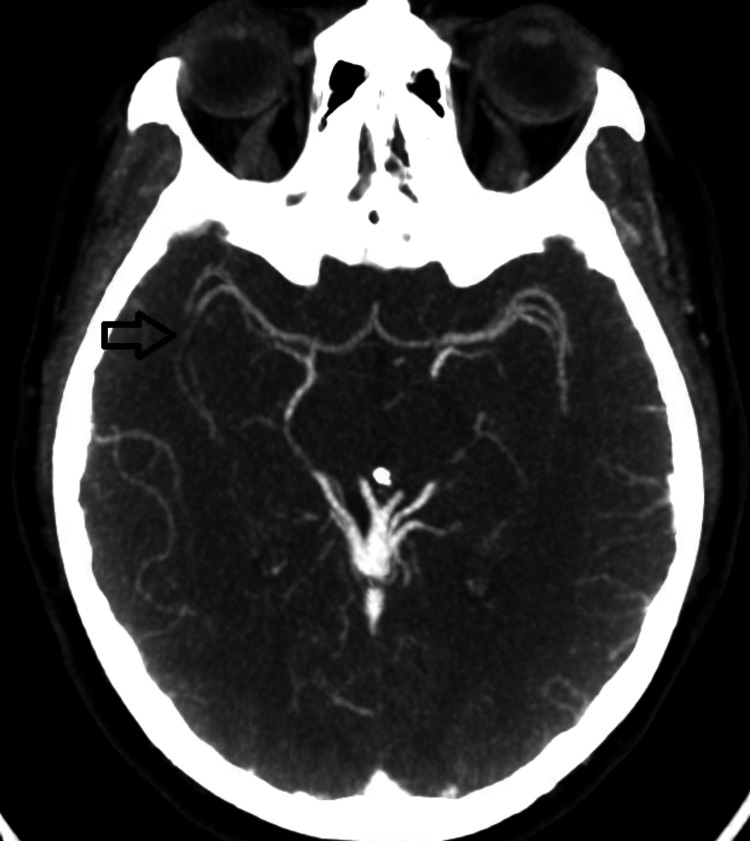
Mild narrowing of the right posterior M2/M3 (arrow)

**Figure 3:**
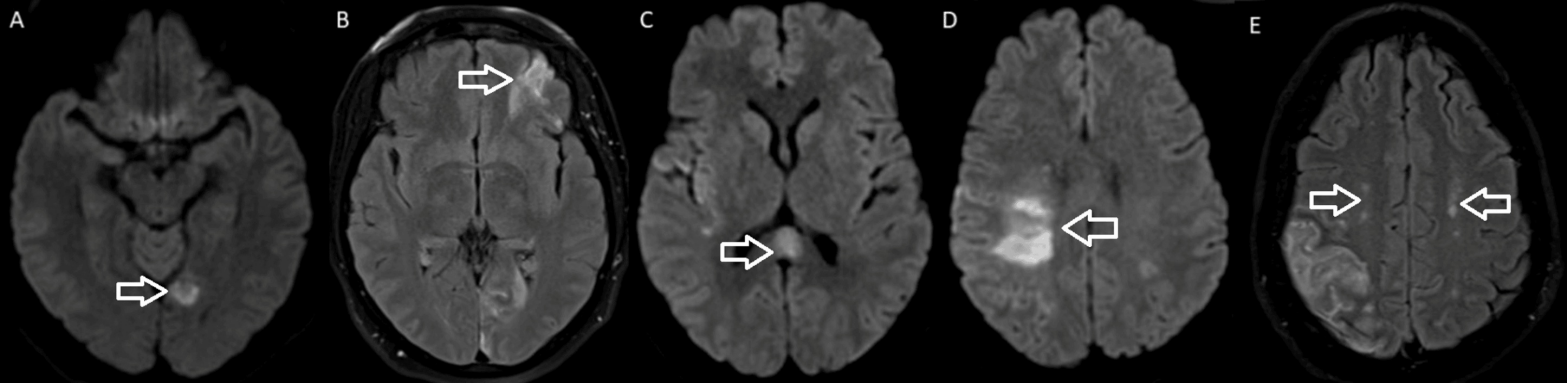
(A) DWI hyperintensity on the left medial occipital (arrow), (B) FLAIR hyperintensity on the left inferior frontal (arrow), (C) DWI hyperintensity on the corpus callosum (arrow), (D) DWI hyperintensity on the right posterior frontal and parietal (arrow), and (E) FLAIR hyperintensity on the bilateral anterior and inferior watershed areas (arrows) DWI: diffusion-weighted imaging, FLAIR: fluid-attenuated inversion recovery

**Figure 4:**
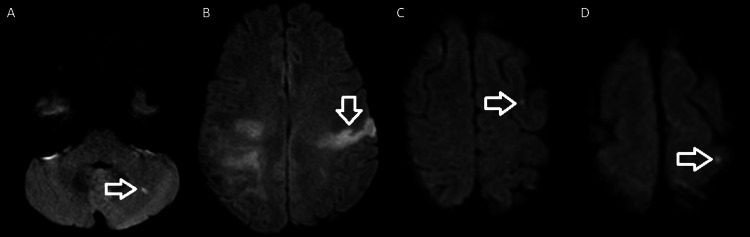
New DWI hyperintensities (arrow) on (A) the left cerebellum, (B) the left precentral gyrus and parietal, and (C, D) the left high frontal

A 42-year-old woman, an active tobacco and marijuana smoker, not on oral contraceptive pills, presented with sudden left hemiplegia. Past medical history was notable for recent cryptogenic right superior middle cerebral artery (MCA) and left posterior pericallosal ischemic stroke with hemorrhagic conversion in the right parietal and left medial occipital lobe. A workup from the recent stroke revealed the following: acute vessel imaging demonstrated irregularity (Figure 1) and mild narrowing of the right posterior M2/M3 MCA branches without large vessel occlusion (Figure 2). MRI demonstrated a left superior MCA encephalomalacia. There is evidence of prior ischemic stroke involving the right superior MCA and posterior pericallosal artery and sequela of prior hemorrhagic conversion involving the right parietal and left medial occipital lobe. In addition, it showed T2 hyperintensities in bilateral anterior and posterior watershed areas (Figure 3). Basic labs (low-density lipoprotein, hemoglobin A1c, and thyroid-stimulating hormone), HIV, beta-human chorionic gonadotropin, stroke workup of the young, and venous Doppler ultrasound on all extremities were normal. CT thorax and CT abdomen pelvis were negative for malignancies. Lumbar puncture and MRI with vessel wall imaging were negative for vasculitis. Her platelet count during the prior admission was 169 and 208 on the day of discharge to rehab. Transthoracic echocardiogram (TTE), transesophageal echocardiogram (TEE), and transcranial doppler (TCD) were significant for grade 3 PFO/intrapulmonary shunt. The presumed etiology was an embolic stroke of an undetermined source. Hence, she was discharged on a single-agent antiplatelet (81 mg aspirin) and high-dose statin (80 mg atorvastatin). She returned 11 days later with new sudden-onset slurred speech and right-sided hemiplegia. Vessel imaging was unchanged from the prior, and repeat MRI showed a new left cortical multifocal anterior and posterior circulation stroke (Figure 4). The suspected mechanism of stroke was cardioembolic, although the patient’s normal left atrial size was presumed to make this mechanism less likely. A few days into her hospital stay, her platelets began to decrease. With new-onset thrombocytopenia (lowest platelet count was 38), an ADAMTS13 screen was ordered and returned positive. The ADAMTS13 activity was <5%, while the ADAMTS13 inhibitor Bethesda titer was notably high (1.4, normal <0.4) (Tables 1-2).

**Table 1:** ADAMTS13 ADAMTS13: a disintegrin and metalloprotease with a thrombospondin type 1 motif, member 13

ADAMTS13	Result	Normal value
ADAMTS13 inhibitor screen	Positive	Negative
ADAMTS13 activity	<5	≥70%
ADAMTS13 inhibitor Bethesda titer	1.4	<0.4

**Table 2:** Complete blood count WBC: white blood cell, RBC: red blood cell

Complete blood count	Result	Normal value
WBC	8.9	4-10 10 x 3/uL
Hemoglobin	12.6	11.5-15.5 g/dL
Hematocrit	36	36-45%
Platelet	38	150-400 10 x 3/uL
RBC	3.78	4.1-5.3 10 x 6/uL

Uncharacteristically, for TTP, the patient did not have a fever, anemia (Hb 12.6), or renal failure (good urine output and Cr 0.68) (Table 3). Nonetheless, given the absence of a clear alternative attributable stroke mechanism, low ADAMTS13 activity, and high ADAMTS13 Bethesda inhibitor titer, TTP was suspected as the etiology of her ischemic strokes. For acute therapy, a three-day 1 mg/kg methylprednisolone was started, and a hematology service was consulted for co-management. The patient completed four rounds of plasmapheresis while receiving 90 mg of prednisone daily. She was then started on a regimen to complete four doses of weekly rituximab. The patient improved clinically during her stay, with noted improvements in platelet count and ADAMTS13 activity.

**Table 3:** Basic metabolic panel BUN: blood urea nitrogen

Basic metabolic panel	Result	Normal value
Sodium	142	136-145 mmol/L
Potassium	3.6	3.4-5.1 mmol/L
Bicarbonate	23	22-29 mmol/L
Chloride	109	98-107 mmol/L
BUN	7	6-20 mg/dL
Creatinine	0.68	0.50-0.90 mg/dL

## Discussion

In a cohort of 17 patients with probable iTTP (low ADAMTS13 activity associated with the presence of ADAMTS13 inhibitors and/or favorable response after immunotherapy), none had all five TTP features. Only 41% of these patients had thrombocytopenia and hemolysis. Similarly, the patient described in our case lacked fever or renal failure. While she did eventually develop thrombocytopenia later in her course, platelet count was normal on admission. This diagnostic challenge is further compounded by atypical presentations, either with the first episode or recurrent symptoms. For instance, there are several cases in the literature whose neurologic manifestations, i.e., ischemic stroke, preceded the hematologic findings [3,8-10]. There are also reports of iTTP with normal hematologic parameters on admission [11-13].

A study involving a large cohort of patients with iTTP who presented with stroke found that smoking, hypertension, old age, and high plasma concentration of ADAMTS13 IgG may contribute to the risk of developing ischemic infarction in these patients [14]. A recent prospective multicenter study on predictors of acute ischemia in patients with TTP or hemolytic uremic syndrome concluded the three variables that predicted the presence of acute ischemic lesions on cerebral MRI were the presence of old infarcts on MRI, the level of blood pulse pressure, and the diagnosis of iTTP [15].

Variable characteristic neuroimaging patterns in patients who present with ischemic stroke related to TTP have been documented. In a retrospective cohort study of 49 patients diagnosed with a first TMA event, imaging tests were performed in 62% of these patients. Imaging detected acute central nervous system lesions such as posterior reversible encephalopathy syndrome, hemorrhagic, and ischemic strokes in 7 (27%) of these patients [16]. Lin et al. [9] analyzed neuroimaging patterns of stroke in a large cohort of patients with iTTP and found that of 108 iTTP patients, 21 had ischemic stroke on neuroimaging. The authors concluded that iTTP patients who can present with large ischemic strokes (defined as strokes >20 mm) are typically younger than patients with small ischemic strokes (<20 mm). While ischemic lesions were apparent on imaging for the aforementioned patients, Zhang and He et al. [1] reported a case of a 65-year-old male who presented with typical acute ischemic stroke symptoms and was diagnosed with TTP. However, his CT and MRI scans were negative for stroke. This patient’s symptoms and lab findings quickly resolved once plasma exchange therapy was initiated. Thus, physicians should consider TTP when a patient presents with acute stroke symptoms and thrombocytopenia despite negative brain CT and MRI.

TTP management includes the expeditious initiation of therapeutics, with the gold standard being TPE [3,17]. Additional management options include plasma infusion and intermediate-purity FVIII concentrates. Novel therapeutics under development for TTP include nanobodies directed against domain A1 of vWF (caplacizumab), recombinant ADAMTS13, plasma cell inhibitors (bortezomib), N-acetylcysteine, and VWF-glycoprotein Ib/IX interaction inhibitors (anfibatide) [18]. Notably, mortality rates have fallen from 90% to 10% since plasma exchange treatment. Since the prompt administration of plasma exchange is related to better outcomes, early diagnosis and treatment are paramount.

Table 4 presents a collection of reported cases in which TTP initially manifested as recurrent multi-territorial ischemic strokes.

**Table 4:** Atypical TTP cases presenting as strokes ADAMTS13: a disintegrin and metalloprotease with a thrombospondin type 1 motif, member 13, CRP: C-reactive protein, ESR: erythrocyte sedimentation rate, FFP: fresh frozen plasma, Hb: hemoglobin, Hct: hematocrit, LDH: lactate dehydrogenase, MCV: mean corpuscular volume, NIHSS: National Institutes of Health Stroke Scale, OTC: over the counter, PBS: peripheral blood smear, PLT: platelet, PCA: posterior cerebral artery, RC: red cells, RUE: right upper extremity, TPE: therapeutic plasma exchange, CTA: computed tomography angiography, MAHA: microangiopathic hemolytic anemia, CT: computed tomography, MCA: middle cerebral artery, MRI: magnetic resonance imaging, MRA: magnetic resonance angiography, MRV: magnetic resonance venography, IV: intravenous, pRBC: packed red blood cell, M: male, F: female, L: left. R: right

Article (year)	Age/sex	Presentation	ADAMTS13 inhibitor	Abnormal labs	Imaging	Management	Days from initial presentation to changes in lab values
Albo et al. (2022) [3]	63/F	Dizziness, confusion, right-sided hemiparesis, sensory deficits, NIHSS 6	ADAMTS13 <5%, elevated ADAMTS13 in 1.5 Bethesda units	Repeat PLT testing showed 8000/uL, PBS showed numerous schistocytes, decreased haptoglobin <8 mg/dL	MRI: acute infarct in L PCA territory involving the L medial temporal lobe, L occipital lobe, and L thalamus; subacute infarcts identified in L cerebellum, L BG, b/l occipital lobes, and R corona radiata; CTA h/n: significant stenosis versus occlusion of P2 of L PCA w/ distal reconstitution	FFP	-
Jameie et al., (2023) [8]	59/M	L hemiparesis, dysarthria, decreased consciousness; over the next 24 hours, the neurological condition deteriorated		Initial labs: low PLT w/o anemia; days later: thrombocytopenia progressed, and MAHA developed; PBS: occasional schistocytes; elevated ESR, CRP, ferritin, fibrinogen, and D-dimer	Brain CT: infarct lesions in R MCA territory; 24 h later CT: new ischemic lesions in L MCA territory; day 8 brain MRI: evidence in favor of subacute infarctions with restriction and mild hemorrhagic transformation in the R parietotemporal, L frontal lobe, and L centrum semiovale; MRA and MRV: no evidence of venous thrombosis or vascular lesions	TPE on day 8, pulse IV methylprednisolone, oral prednisolone, rituximab added d/t refractory course of the disease	PLT count reached 189* at discharge (48 days of hospitalization); during the follow-up, the neurological symptoms progressively improved, and he did not experience further relapses or report adverse effects
Badugu and Idowu (2019) [12]	56/M	L-sided weakness, slurred speech, L facial droop	ADAMTS13 <5%, residual ADAMTS13 activity <0.4 inhibitor units	Initial PLT 115,000, LDH 794, Hb 10.7, Hct 31.2, RC 2.8%, PBS: 5-6 schistocytes/high power field		Thrombolytic therapy showed mild improvement, TPE	Day 3: PLT 167,000 per cubic mm
Filatov et al. (2020) [19]	31/F	Garbled speech, paresthesias	ADAMTS13 <5%	PLT 4,000, LDH 1,154, Hb 6.6, MCV 93; PBS: schistocytes present	CT head negative for acute IC findings, MRA negative for embolic event	FFP, pRBC, plasmapheresis, rituximab, plasmapheresis, prednisone	Platelet count of just over 200,000 and Hb of 10.2 after a 36-day hospital stay
Ramesh et al. (2023) [20]	61/M	RUE weakness, intermittent frontal headaches, usually resolving with OTC analgesics	ADAMTS13 <5%, anti-ADAMTS13 antibody was mildly elevated at 18 × 103 U/L	Initial labs: mild anemia (Hb 117 g/L), PLT 98 x 10^9, Cr 1.6 mg/dL, low haptoglobin <3 umol/L, LDH 537 IU/L, reticulocytosis 2.3%, PBS 0-2 schistocytes per HPF	CT brain: remote infarcts, MRI: multiple, disseminated, small, peripheral, acute-to-early subacute infarcts	Initial tx: aspirin, clopidogrel, and statin; after TTP diagnosis: prednisone, plasmapheresis, and rituximab; discharged with prednisone, rituximab, and a prophylactic antibiotic	-

## Conclusions

TTP is a rare disorder, and variations in presenting symptoms can represent a diagnostic challenge, as reflected in this case report. The neurologic manifestation secondary to multifocal recurrent stroke preceded the hallmark manifestation of TTP, thrombocytopenia. Importantly, thrombocytopenia may appear until later in the disease course with variant presentations of TTP. A low threshold to consider atypical etiologies when pursuing workup for cryptogenic stroke should be in mind when evaluating young adults with recurrent multi-territory ischemic stroke.
